# Soret and Dufour influences on forced convection of Cross radiative nanofluid flowing via a thin movable needle

**DOI:** 10.1038/s41598-022-23563-5

**Published:** 2022-11-04

**Authors:** M. Israr Ur Rehman, Haibo Chen, Aamir Hamid, Sajid Qayyum, Wasim Jamshed, Zehba Raizah, Mohamed R. Eid, El Sayed M. Tag El Din

**Affiliations:** 1 School of Mathematics and Statistics, Central South University, Changsha, 410083 China; 2 Department of Mathematics, Women University of Azad Jammu and Kashmir, Bagh, 12500 Azad Kashmir Pakistan; 3 Department of Mathematics, University of Mianwali, Mianwali, Pakistan; 4 Department of Mathematics, Capital University of Science and Technology (CUST), Islamabad, 44000 Pakistan; 5 Department of Mathematics, College of Science, King Khalid University, Abha, Saudi Arabia; 6 Department of Mathematics, Faculty of Science, New Valley University, Al-Kharga, 72511 Al-Wadi Al-Gadid Egypt; 7 Department of Mathematics, Faculty of Science, Northern Border University, Arar, 1321 Saudi Arabia; 8 Electrical Engineering, Faculty of Engineering and Technology, Future University in Egypt, New Cairo, 11835 Egypt

**Keywords:** Mathematics and computing, Physics

## Abstract

The main feature of the current investigation is to analyze the magnetohydrodynamic mixed convection flow of Cross fluid. Flow is due to a movable thin needle with Soret and Dufour effect. Heat generation/absorption and nonlinear heat radiation are used in the energy equation. Characteristics of the chemical reaction and thermal activation are given special attention. Appropriate variables are introduced for the transformation of partial differential equations to ordinary differential equations. With the assistance of Runge–Kutta Fehlberg's fourth- fifth-order method with the shooting technique, we determined the prominent result numerically. The prominent examined parameters range is velocity and temperature ratios, heat generation, Dufour, Hartmann, Schmidt numbers ($0.1\le{{\lambda}},{{{\theta}}}_{{{w}}},{{Q}},{{{D}}}_{{{u}}},\boldsymbol{ }{{M}},{{S}}{{c}}\le 0.7$), needle thickness ($0\le {{a}}\le 15$), radiative parameter ($5\le {{R}}{{d}}\le 8$), and Weissenberg number ($0.01\le {{W}}{{e}}\le 0.09$), respectively. Graphs for velocity, thermal, concentration, Skin friction coefficient, and heat and mass transport rates are displayed and analyzed for physical parameters. A similar observation of mixed convection and needle thickness parameter is seen on the velocity field. Temperature and heat transfer rate are reverse behavior in the frame of the Dufour effect. Moreover, an enhancement in chemical reaction shows decay to the concentration field.

## Introduction

Viscosities of the numerous liquids we come across every day are affected by the applying shear force. Although, there is various type of liquid that exhibits fundamentally different performance. Several of these fluids are shear-thicker, which means that the relation between their viscosities and the strain rate is inverse. Such application is exhibited in almost all mayonnaise, colloidal suspensions, polymer melts and solution, biological liquids, etc. A simplified two-effect (power-law) scheme, introduced by Oustwald^1^ and well clarified by Reiner^2^ has gained comprehensive discussion in the research for inelastic non-Newtonian liquids. This framework offers the shear rate curve and shear stress for a limited variety of shear rates. Munzar et al.^3^ present an investigation of heat radiative and convective boundary condition impacts on a mixed convection thermal transport for the passage Cross liquid along a vertical stretched sheet into consideration of buoyancy impacts. Tochi^4^ deduced analytic formulas connecting volumetric stream rate to pressured decline in circular tubes utilizing both the Cross-Carreau liquid schemes. The boundary layer computations for 2-dimensional stream and Cross-liquid energy transport passing through the linear stretched sheet were provided by Khan et al.^5^. Their research’s conclusion depicts that enhancing the power-law effect the thicknesses of the heated boundary layer. Khan et al.^6^ investigated a liquid stream around a radial extending disc embedded in a resting fluid satisfying a Cross-fluid scheme utilizing a computational method. Additionally, the most impressive current works are displayed in^7–10^.

Flow across a thin needle has piqued the attention of investigators who expected its use in industrial products such as electrical devices, hot wire anemometers, and geothermal power generation. The thinner needle is identified as a body of change with a thickness smaller than that of the boundary layer. Lee^11^ was the first to explore the flow of viscous liquid toward a thinner needle. The researchers next investigated this work by taking into consideration numerous physical aspects^12–15^. Mabood et al.^16^ assessed the thermal management of the Cross-micropolar fluids stream over a thinner needle focusing on Arrhenius activation thermal and binary chemical reaction. Khan et al.^17^ studied the heat transport for the stream of nanoliquid toward a thinner hot needle with the impacts of Hall current and viscous dissipation. Xiong et al.^18^ examined the 2-dimensional, steady, laminar, and incompressible magneto-Cross nanoliquid flow across a movable vertical thinner needle with the incidence of chemical reaction, Darcy–Forchheimer permeable media, mixed convection, Ohmic, and viscous dissipation.

When thermal and mass transport appears concurrently in a changing liquid, the relationships between the fluxes and drive potentials become increasingly complex. It has been revealed that thermal fluxes may be produced not only by thermal estimation but also by concentric estimation. The diffusion-thermos (Dufour) effect describes heat transmission initiated by a concentric gradient. The mass transport produced by a temperature distribution, on the other hand, is known as the thermal-diffusive (Soret) influence. Such impacts are significant in hydrology, nuclear waste management, petrology, geothermal energy, nuclear reactors, oil reservoirs, groundwater pollutant migration, manufacture of rubber and plastic sheets, isotopes separation, the mixture of gases, and compact heat insulation exchangers. The consequences of the Dufour and Soret influences amalgamated with the Hartmann field on a Casson fluid across a stretchable surface are addressed by Hayat et al.^19^. Hayat et al.^20^ explored the influences of, Soret and Dufour parameters on 3-dimensional second-grade MHD liquid stream through an exponentially radiative stretchable surface. Farooq et al.^21^ investigated the incompressible electrically conducting Oldroyd-B liquid flow through a stretchable surface in the existence of Dufour and Soret impacts. Shojaei et al.^22^ analytically assessed the stream of non-Newtonian liquid stream through a cylinder subjected to the thermal radiations, Soret, and Dufour consequences. Waini et al.^23^ demonstrated the influences of Soret and Dufour and diffusive on the flow of Al_2_O_3_-water nanoliquid along a thinner needle by considering the Tiwari-Das model. Rasool et al.^24^ studied the outcomes of thermal radiation, chemical reaction, and Soret–Dufour influences on an incompressible steady Darcy–Forchheimer nanofluids flow along a stretchable surface. Alzahrani et al.^25^ examined 3-dimensional, magnetohydrodynamic (MHD) embraced flow in a permeable rotating channel subject to viscous dissipation, applied magnetic field, Dufour and Soret impacts.

The minimal amount of energy needed to start a chemical reaction is identified as thermal activation. This concept of activation energy was first announced by Svante Arrhenius in the year 1889. In free convection boundary layer flows, thermal activation along with mass and heat transport has a considerable role. Thermal activation is particularly imperative in the sectors of the oil reservoir, mechanic chemistry, water emulsions, chemical engineering, geothermal industrial, and food management. Numerous researchers investigated the performance of activation energy in different media: Hayat et al.^26^ studied Ree–Eyring nanoliquid flow with Arrhenius activation energy and entropy optimization between two rotating surfaces. Kalaivanan et al.^27^ analyzed the influences of thermal activation on flow heat and mass transport features of second-grade nanoliquid over a stretching surface. Muhammad et al.^28^ investigated the flow of Eyring–Powell nanoliquid with the consideration of modified thermal and mass fluxes, nonlinear heat radiative, velocity slip condition, and Arrhenius activation energy. Several recent attempts regarding activation energy can be seen through studies^29–40^.

The peculiarity of this research is that it investigates the Cross-diffusion influence on the mixed convection stream of magnetized Cross liquid toward a vertical movable thin needle with Soret and Dufour variation. The governing system of flow mechanism is transformed into ordinary differential equations employing similarity transmission and then solved numerically by Runge–Kutta Fehlberg's fourth- fifth-order technique with a shooting scheme. The projections of developing parameters on drag coefficient, heat flux, and Sherwood numbers, with movement, thermal, and concentric curve are revealed in graphs and debated.

## Mathematical formulation

**Figure 1:**
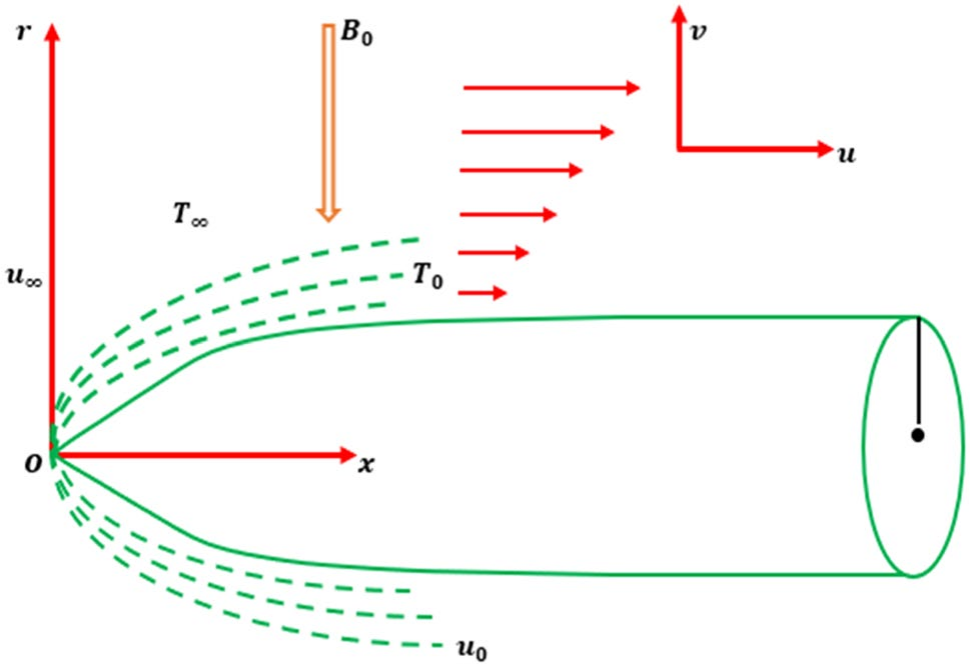
Flow geometry.

In this analysis, in the occurrence of Soret and Dufour variation, we examined an incompressible laminar boundary layer stream of a Cross nanoliquid across a movable thinner needle. Figure 1 shows a schematic illustration of the prominent scheme and coordinate system.

The axial and radial coordinates are $x$ and $r$, respectively, while the radius of the needle is $r = R(x)$. The needle leading edge is measured vertically on the *x*-axis, while $r$ is always perpendicular to the x-axis. The influence of the needle's transverse curvature is essential since it is thin, but the pressure gradient toward the surface can be ignored. We supposed that the needle surface thermal is ${T}_{w}$, with ${T}_{w}$ > T indicating a heated needle (aiding flow) and ${T}_{w}$ < T indicates a cooled needle (opposed flow), and that the surface volume fraction is ${C}_{w}>C$. Owing to the minimum magnetic Reynolds number, the Hartmann field ${B}_{0}$ is utilized parallel to the flow direction, and the generated Hartmann field can be neglected. The needle's movable constant velocity ${U}_{w}$, in a similar or opposing direction as a constant velocity U free stream flow. Additionally analyzed that the needle’s size is thinner so that the pressure gradient is negligible while transverse curvature has a certain impact. The boundary layer description for mass, momentum, heat energy and solid volume fraction can be stated as follows under the preceding assumptions^13,17,41^:

1 $$\frac{\partial }{\partial x}\left(ru\right)+\frac{\partial }{\partial r}\left(rv\right)=0$$

2 $$\left.\begin{array}{c}u\frac{\partial u}{\partial x}+v\frac{\partial u}{\partial r}={v}_{f}\left(\frac{\partial }{\partial r}+\frac{1}{r}\right)\left[\left(\frac{\partial u}{\partial r}\right)\left\{1+\left(\Gamma {\left|\frac{\partial u}{\partial r}\right|}^{n}\right)\right\}\right]-\frac{\sigma {B}_{0}^{2}u}{\rho }\\ +\frac{1}{{\rho }_{f}}\left[\left(1-{C}_{\infty }\right){\rho }_{f\infty }{B}_{T}\left(T-{T}_{\infty }\right)-\left({\rho }_{p}-{\rho }_{f\infty }\right)\left(C-{C}_{\infty }\right)\right]g\end{array}\right\}$$

3 $$u\frac{\partial T}{\partial x}+v\frac{\partial T}{\partial r}=\frac{K}{{\rho }_{f}{C}_{p}}\frac{1}{r}\frac{\partial }{\partial r}\left[\left(1+\frac{16{\sigma }_{s}}{3{k}_{e}{k}_{f}}{T}_{\infty }^{3}\right)\left(r\frac{\partial T}{\partial r}\right)\right]+\frac{{D}_{m}{K}_{T}}{cs}\frac{1}{r}{\left(r{C}_{r}\right)}_{r}$$

4 $$u\frac{\partial C}{\partial x}+v\frac{\partial C}{\partial r}=\frac{D}{r}\frac{\partial }{\partial r}\left(r\frac{\partial C}{\partial r}\right)+\frac{{D}_{m}{K}_{T}}{{T}_{m}}\frac{1}{r}{\left(r{T}_{r}\right)}_{r}-{K}_{r}^{2}\left(C-{C}_{\infty }\right){\left(\frac{T}{{T}_{\infty }}\right)}^{n}{e}^{\frac{{E}_{0}}{{K}^{*}T}}$$

The associated boundary condition is^16^:

5 $$\begin{gathered} u = U_{w} ,v = 0,T = T_{w} ,C = C_{w} ,r = R\left( x \right), \hfill \\ u \to U_{\infty } ,T \to T_{\infty } ,C \to C_{\infty } ,asr \to \infty \hfill \\ \end{gathered}$$

where $u, v, T, n, {\rho }_{f}, {T}_{\infty }, C, {\rho }_{p}, \sigma , {v}_{f}, {C}_{\infty }, \mu , {C}_{p}, {D}_{m}, g, {C}_{s}, {K}_{r}^{2}$ and $Kr$ represents radial and axial velocity components, temperature, power law index, the density of fluid, ambient temperature, concentration, the density of nanoparticles, electric conductivity, kinematics viscosity, ambient concentration, dynamic viscosity, heat capacity, mass diffusivity, thermal expansion coefficient, concentration susceptibility, chemical reaction constant and chemical response parameter respectively.

## Nondimensional solve

The similarity transmission is^16^:

6 $$\eta = \frac{{U_{0} r^{2} }}{{v_{f} x}}, \psi = v_{f} xf\left( \eta \right), \theta \left( \eta \right) = \frac{{T - T_{\infty } }}{{T_{w} - T_{\infty } }}, \phi \left( \eta \right) = \frac{{C - C_{\infty } }}{{C_{w} - C_{\infty } }}$$

where Eq. (1) is assured identically, and Eqs. (2–5) are interpreted into

7 $${R}_{e}\left[1+\left(1-n\right){\left(4We{f}^{{{\prime}}{{\prime}}}\right)}^{n}\right]{f}^{{{\prime}}{{\prime}}{{\prime}}}+\frac{1}{2}{\left[1+{\left(4We{f}^{{{\prime}}{{\prime}}}\right)}^{n}\right]}^{2}+f{f}^{{{\prime}}{{\prime}}}-M{f}^{{\prime}}+\frac{{\lambda }_{1}}{4}\theta -\frac{Nr}{4}\phi =0$$

8 $$\frac{1}{Pr{{\left[\left(1+Rd{\left(1+\left({\theta }_{w}-1\right)\theta \right)}^{3}\right)\left(\eta {\theta }^{{\prime}}\right)\right]}^{{\prime}}}^{{\prime}}\frac{1}{2}\frac{{D}_{u}}{2}\left(\eta {\phi }^{{{\prime}}{{\prime}}}+{\phi }^{{\prime}}\right)}$$

9 $$2\eta {\phi }^{{{\prime}}{{\prime}}}+2{\phi }^{{\prime}}+ScSr\left(\eta {\theta }^{{{\prime}}{{\prime}}}+{\theta }^{{\prime}}\right)+\frac{Sc}{2}f{\phi }^{{\prime}}-\frac{1}{2}\Gamma Sc\phi {\left(1+\delta \theta \right)}^{n}\mathit{exp}\left(\frac{-E}{1+\delta \theta }\right)=0$$

10 $$\begin{gathered} f\left( a \right) = \frac{\lambda }{2}a,\theta \left( a \right) = 1,f^{\prime}\left( a \right) = \frac{\lambda }{2},\phi \left( a \right) = 1, \hfill \\ \theta \left( \eta \right) \to 0,f^{\prime}\left( \eta \right) \to \left( {\frac{1 - \lambda }{2}} \right),\phi \left( \eta \right) \to 0\,\,at\,\,\eta \to \infty \hfill \\ \end{gathered}$$

where the dimensionless number in Eqs. (7–10) are $Re$, ${\lambda }_{1}$, $Pr$, $We$, ${D}_{u}$, $Rd$, $a$, $M$, $Nr$, $Sc$, $E$, ${\theta }_{w}$, $\Gamma$, $\lambda ,$ and $Sr$ represent Reynolds number, mixed convection factor, Prandtl number, Weissenberg number, Dufour number, radiation parameter, needle thickness variable, Hartmann number, buoyance ratio parameter, Schmidt number, activation energy parameter, temperature ratio parameter, chemical reaction parameter, heat generation/absorption variable, velocity ratio parameter, and Soret number via, are express below.

11 $$\left.\begin{array}{c}We=\frac{{u}_{w}{R}_{e}}{U},\lambda =\frac{G{r}_{x}}{{R}_{e}},G{r}_{x}=\frac{\left(1-{C}_{\infty }\right)\left({T}_{w}-{T}_{\infty }\right)g{B}_{T}}{{v}^{2}},M=\frac{\sigma {B}_{0}^{2}x}{2\rho U},\\ Nr=\frac{\left({\rho }_{p}-{\rho }_{f\infty }\right){C}_{\infty }gx}{{U}^{2}{\rho }_{f}},\mathit{Pr}=\frac{\rho {C}_{p}v}{K},Rd=\frac{16{\sigma }_{s}{T}_{\infty }^{3}}{3{k}_{e}{k}_{f}},Q=\frac{{Q}_{0}x}{\rho {C}_{p}U},\\ Sc=\frac{{v}_{f}}{D},{\theta }_{w}=\frac{{T}_{w}}{{T}_{\infty }},Sr=\frac{\left({T}_{w}-{T}_{\infty }\right){D}_{m}{K}_{T}}{\left({C}_{w}-{C}_{\infty }\right){T}_{m}\mu {C}_{p}},\Gamma =\frac{x{K}_{r}^{2}}{U},\lambda =\frac{{U}_{w}}{U},\\ {R}_{e}=\frac{{u}_{x}}{v},\delta =\frac{{T}_{w}-{T}_{\infty }}{{T}_{\infty }},{D}_{u}=\frac{\left({C}_{w}-{C}_{\infty }\right){D}_{m}{K}_{T}}{\left({T}_{w}-{T}_{\infty }\right)\left(cs\mu {C}_{p}\right)},E=\frac{{E}_{0}}{K{T}_{\infty }}\end{array}\right\}$$

Here ${C}_{fx}$, ${Nu}_{x}$ and ${Sh}_{x}$ denotes friction factor coefficient, heat flux, and mass flux correspondingly.

12 $$\left.\begin{array}{c}{C}_{f}=\frac{2\mu }{\rho {U}_{w}^{2}}{\left\{1+{\left(\Gamma \left|\frac{\partial u}{\partial r}\right|\right)}^{n}\right\}}^{-1}{\left(\frac{\partial u}{\partial r}\right)}_{r=R},\\ N{u}_{x}=\frac{x}{K\left({T}_{w}-{T}_{\infty }\right)}{\left(\frac{\partial T}{\partial r}\right)}_{r=R}+qr,\\ S{h}_{x}=\frac{-x}{\left({C}_{w}-{C}_{\infty }\right)}{\left(\frac{\partial C}{\partial r}\right)}_{r=R}\end{array}\right\}$$

In dimensionless form:

13 $$\left. {\begin{array}{*{20}c} {\sqrt[{f_{x}^{\frac{1}{2}} \left[ {\frac{{f^{\prime\prime}\left( a \right)}}{{\left( {1 + We\left| {f^{\prime\prime\prime}\left( a \right)} \right|^{n} } \right)}}} \right]}]{Re}} \\ {Nu_{x} {\text{Re}}_{x}^{ - 1/2} = - 2a^{\frac{1}{2}} \left( {1 + Rd\left( {1 + \left( {\theta_{w} - 1} \right)\theta \left( a \right)} \right)^{3} } \right)\theta^{\prime}\left( a \right),} \\ {Sh_{x} {\text{Re}}_{x}^{ - 1/2} = - 2a^{\frac{1}{2}} \phi^{\prime}\left( a \right)} \\ \end{array} } \right\}$$

where ${Re}_{x}$ = $\frac{{u}_{w}}{{v}_{f}}x$ depicts the local Reynolds number.

## Numerical procedure

Due to the difficulty of obtaining an analytic solution for higher-order nonlinear ODEs, a suitable numerical technique with great convergence exactness must be adopted for this system. The numerical findings are obtained by RK-fourth order and shooting techniques.

Equations (1)–(4) along with Eq. (5) are solved with the combination of RK-fourth order and shooting techniques.

By utilizing $$h_{1} = f, h_{2} = f^{\prime}, h_{3} = f^{\prime\prime}, h_{4} = \theta , h_{5} = \theta^{\prime}, h_{6} = \phi , h_{7} = \phi^{\prime}$$

Equations (7)–(10) can be analyzed as per the rules:

14 $$\left. {\begin{array}{*{20}l} {h_{1} = h_{2} ,} \hfill \\ {h_{2} = h_{3} ,} \hfill \\ {h_{3} = \frac{1}{{{\text{Re}} \left[ {1 + (1 - n)(4Weh_{2} )^{n} } \right]}}\left[ {\frac{1}{2}\left[ {1 + (4Weh_{2} )^{n} } \right]^{2} + h_{1} h_{2} + \frac{{\lambda_{1} }}{4}h_{4} - \frac{Nr}{4}h_{6} } \right],} \hfill \\ {h_{4} = h_{5} ,} \hfill \\ {h_{5} = - \frac{\Pr }{{\left( {1 + Rd(1 + (\theta_{w} - 1)h_{4} )^{3} } \right)}}\left[ {\begin{array}{*{20}c} {\frac{1}{\Pr }\left[ {3\eta Rd(\theta_{w} - 1)(1 + (\theta_{w} - 1)h_{4} )^{2} h_{5} } \right] + h_{1} h_{5} } \\ { + \frac{1}{2}Qh_{4} + \frac{{D_{u} }}{2}\left( {\eta h_{7} + h_{7} } \right)} \\ \end{array} } \right]} \hfill \\ {h_{6} = h_{7} ,} \hfill \\ {h_{7} = - \frac{1}{2\eta }\left[ {2h_{7} + ScSr(\eta h_{5} + h_{5} ) + \frac{Sc}{2}h_{1} h_{7} - \frac{1}{2}\Gamma Sch_{6} (1 + \delta h_{4} )^{n} \exp \left( {\frac{ - E}{{1 + \delta h_{4} }}} \right)} \right],} \hfill \\ \end{array} } \right\}$$

with

15 $$\left.\begin{array}{c}{h}_{1}\left(a\right)=\frac{\lambda }{2}a,{h}_{4}\left(a\right)=1,{h}_{2}\left(a\right)=\frac{\lambda }{2},{h}_{6}\left(0\right)=1,\\ {h}_{2}\left(\infty \right)\to \left(\frac{1-\lambda }{2}\right),{h}_{4}\left(\infty \right)\to 0,{h}_{6}\left(\infty \right)\to 0.\end{array}\right\}$$

**Figure 2:**
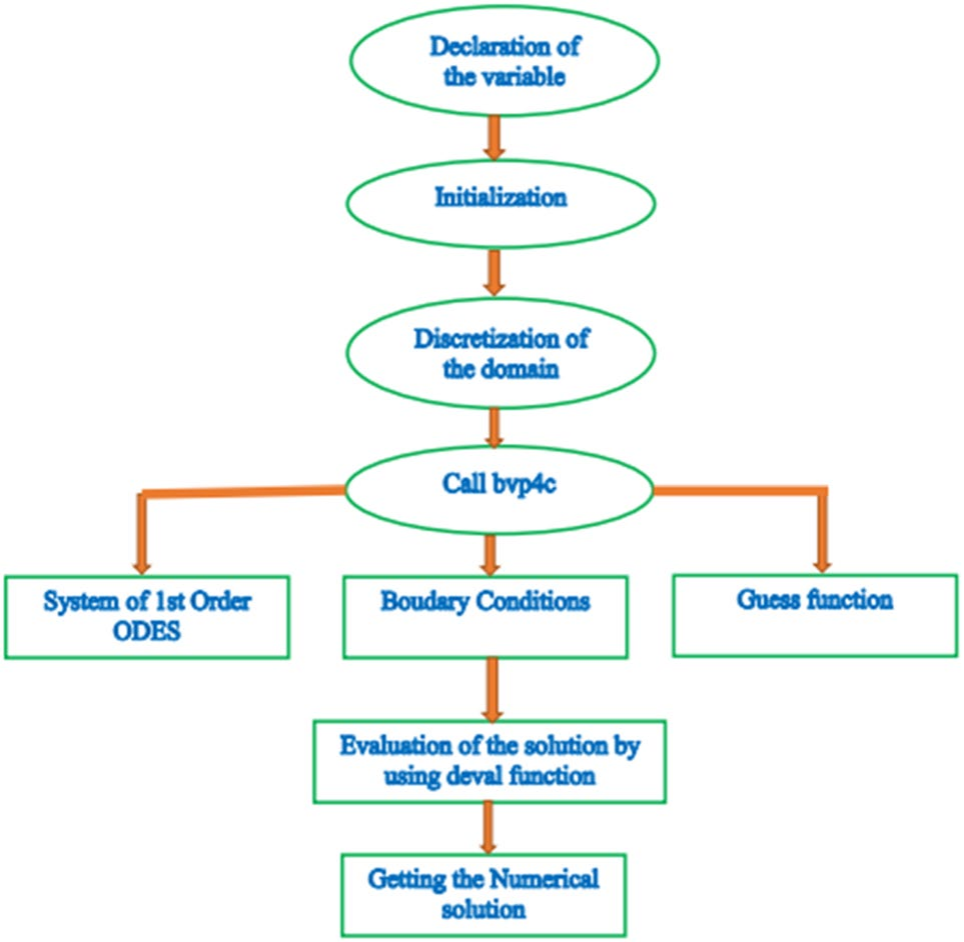
The flow chart of the numerical procedure.

In this development, the existence of a continuous outcome stimulates the grid choice and error function. The margin remains fixed at ${10}^{-4}$. The valuation of $\eta \to \infty$ implies that under this approach, each number response reaches asymptotic characteristics perfectly. The detailed flow chart has also been provided for a clearer and better comprehension of the existing shooting technique (see Fig. 2).

## Code validity

Table 1 is analyzed the comparison of the skin friction coefficient with the previously published article. It is observed that the present work is in good agreement with the preceding result in the limiting sense.

**Table 1:** Comparison of $f^{\prime\prime}\left( 0 \right)$ with Makinde et al.^42^ for numerous valuations of $M$ when $n=We=Nr=Re={\lambda }_{1}=0$.

$M$	Ref.^42^	Present
0.0	− 1.17372	− 1.17365
0.2	–	− 1.19004
0.4	–	− 1.23805
0.6	–	− 1.31454
0.8	–	− 1.41526
10	− 1.53571	− 1.53570

## Results and discussion

**Figure 3:**
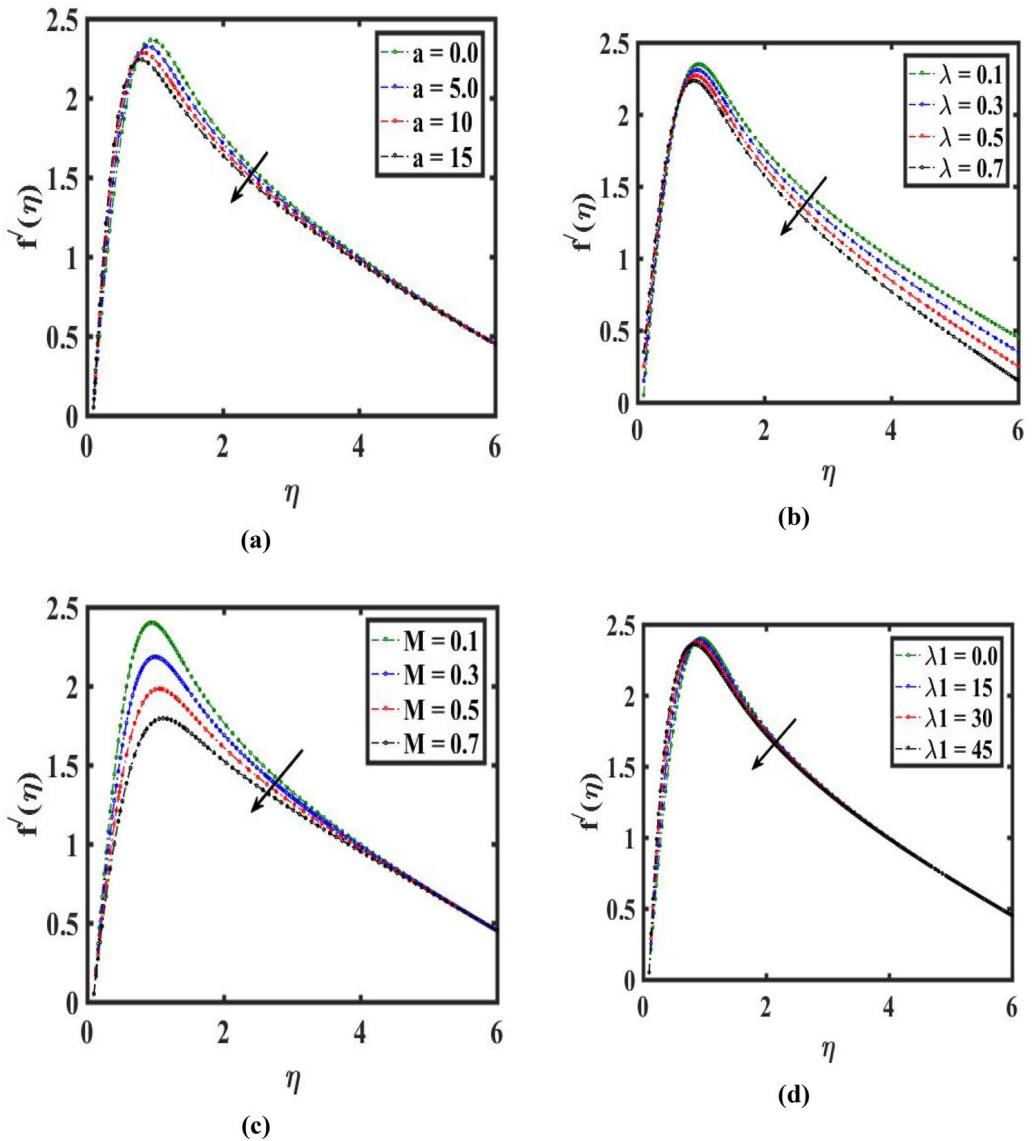
Variation of needle thickness $a$, velocity ratio $\lambda$, Hartmann number $M$, and mixed convection ${\lambda }_{1}$ on velocity ${f}^{{\prime}}(\eta )$.

**Figure 4:**
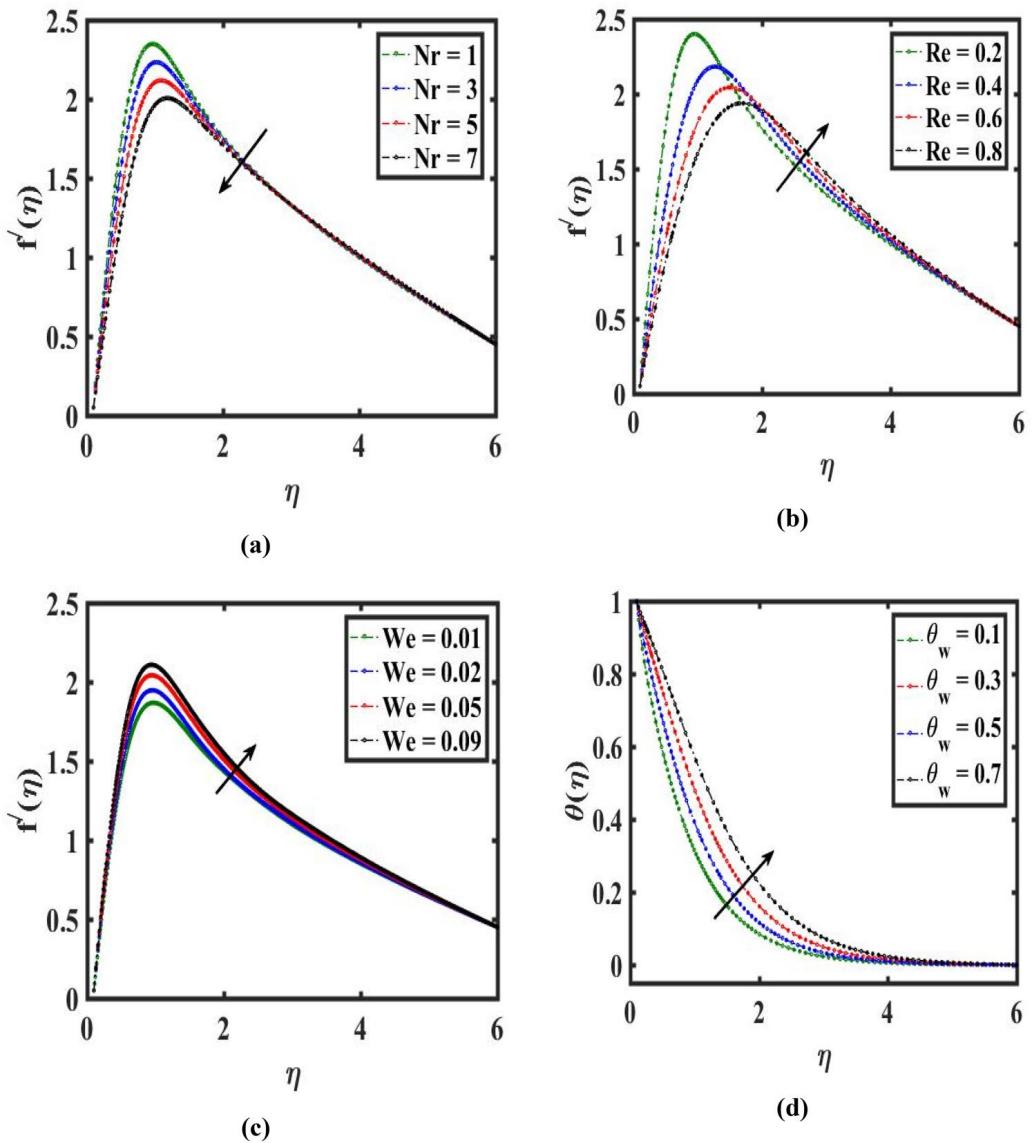
Impact of buoyance ratio $Nr$, Reynolds number $Re$, Weissenberg number $We,$ and temperature ratio ${\theta }_{w}$ on velocity ${f}^{{\prime}}(\eta )$ and temperature $\theta (\eta )$.

The present explanation illustrated the properties of comparative evaluation, velocity curve, heat flux, thermal field, mass flux, concentration estimation, and drag coefficient utilizing graphically and computed numerically. Figure 3a,b reveals the characteristics of the velocity curve ${f}^{{\prime}}(\eta )$ for various needle thickness parameter $(a)$ and velocity ratio parameter $(\lambda )$. It is well known that improving $(a)$ and $(\lambda )$ decline the velocity curve ${f}^{{\prime}}(\eta )$ and momentum of liquid. Infect for a higher valuation of the thinner needle parameter the area of the needle is increase-es so the contact needle surface area with fluid increases which produces higher resistance to the liquid motion. Therefore, the velocity profile decreases. Figure 3c illustrates the stream controlling of the $(M)$ on the ${f}^{{\prime}}(\eta )$. In this case, improving $(M)$ decays the momentum and velocity of the liquid. Actuality, it has been viewed that the presence of the Hartmann field in the flowing field area declines the liquid movement. This analysis demonstrated that the Hartmann force adds struggle to the flow which lowers its velocity. Figures 3d, 4a are presented to demonstrate the impact of $({\lambda }_{1})$ and $(Nr)$. It should be noticed that has been dropped in order to improve the amount of $({\lambda }_{1})$. In addition, the $({\lambda }_{1})$ is associated with buoyance forces that restrict the liquid velocity from growing. As an outcome, for greater $({\lambda }_{1})$ liquid velocity drops. It is also discovered that the velocity curve diminishes by changing the estimation of $(Nr)$. The influence of $({R}_{e})$ and $(We)$ on the movement of liquid molecules are depicted in Fig. 4b,c. liquid velocity and associated layer thicker raises are noticed for improving assessment of $({R}_{e})$. It is also demonstrated that with bigger estimates of $(We)$, velocity climbs. Pertinently, an increment in time of relaxing leads to an augmented in ${f}^{{\prime}}(\eta )$.

**Figure 5:**
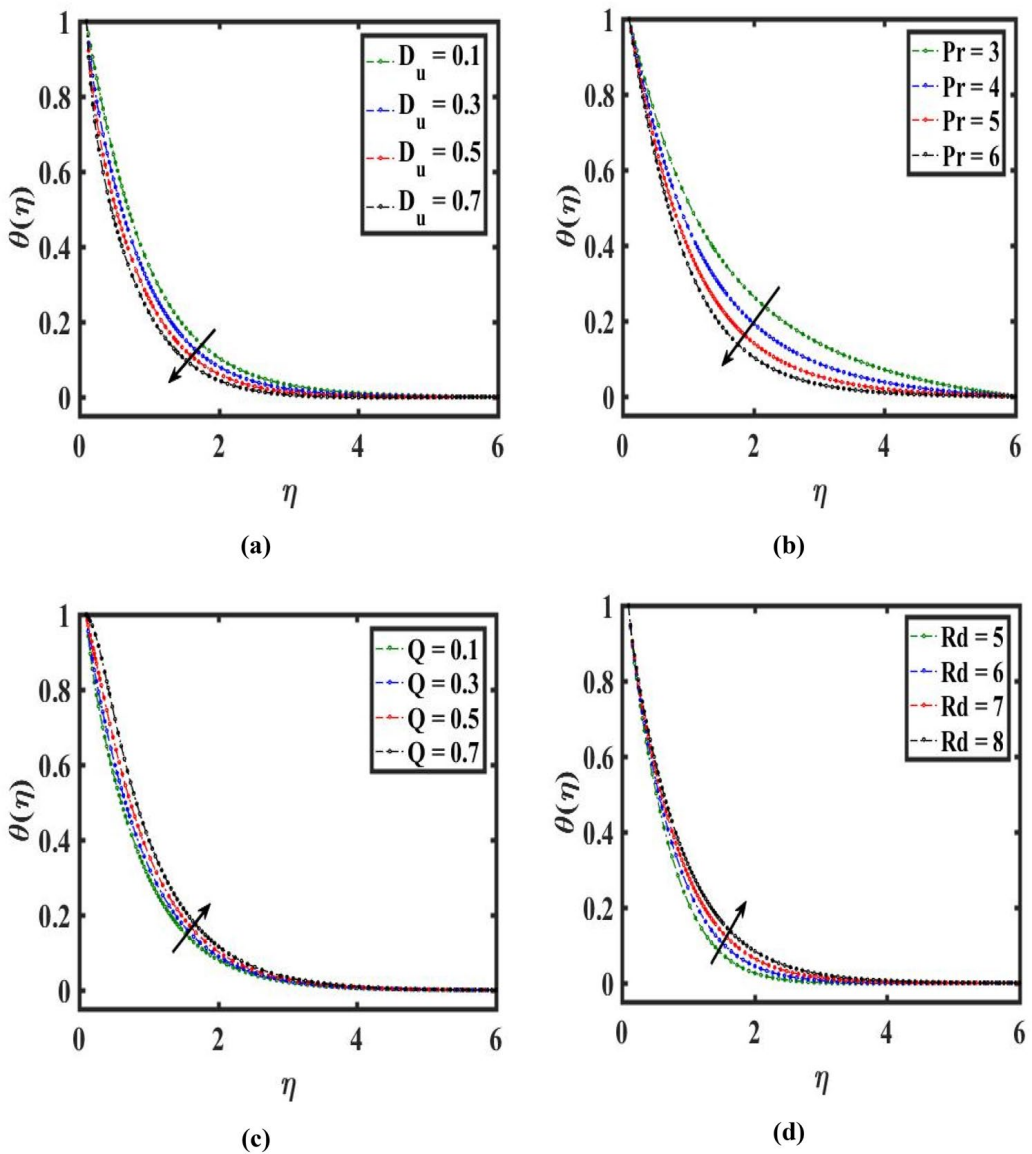
Effect of Dufour number ${D}_{u}$, Prandtl number $Pr$, heat generation $Q$ and radiation $Rd$ on temperature $\theta \left(\eta \right).$

The variance of $\theta (\eta )$ for distinct estimation of $({\theta }_{w})$ and $({D}_{u})$ is highlighted in Figs. 4d, 5a. we can deduce $\theta (\eta )$ from these figures that climb for growth $({\theta }_{w})$ but the reverse pattern can be observed for $({D}_{u})$. Basically, a greater scale of $({\theta }_{w})$ represent larger melting thermal as compared to ambient liquid thermal. As an outcome, $\theta (\eta )$ improves. On augmenting in $({D}_{u})$ thermal curve $\theta (\eta )$ decays which outcomes in heat transport. Thus, a physical improvement is investigated in the temperature estimation $\theta (\eta )$ which is very significant for the stretchable needle.

Figure 5b provides a variety of temperatures because of $(Pr)$. Since $(Pr)$ corelates between the momentum and thermal diffusivities. Here larger $(Pr)$ leads to low thermal diffusivity which shows decay temperature. Figure 5c displays the behavior of heat generating and absorbing variable $(Q)$ on $\theta (\eta )$. It is observed that when $(Q)$ is boosted, a large estimation of thermal is produced. As a result, the mechanisms of higher heat and hence the thermal curve $\theta (\eta )$ escalate. Figure 5d highlights the tendency of the boundary-layered stream field curve towards the variation in the amplitude of $(Rd)$. It is clear from the figure that has a greater influence of $(Rd)$ on thermal estimation $\theta (\eta )$. It is demonstrated that the thermal curve $\theta (\eta )$ is optimized as $(Rd)$ upsurges. This assumption supports the fact that $(Rd)$ is important for delivering additional heat in the liquid stream process.

**Figure 6:**
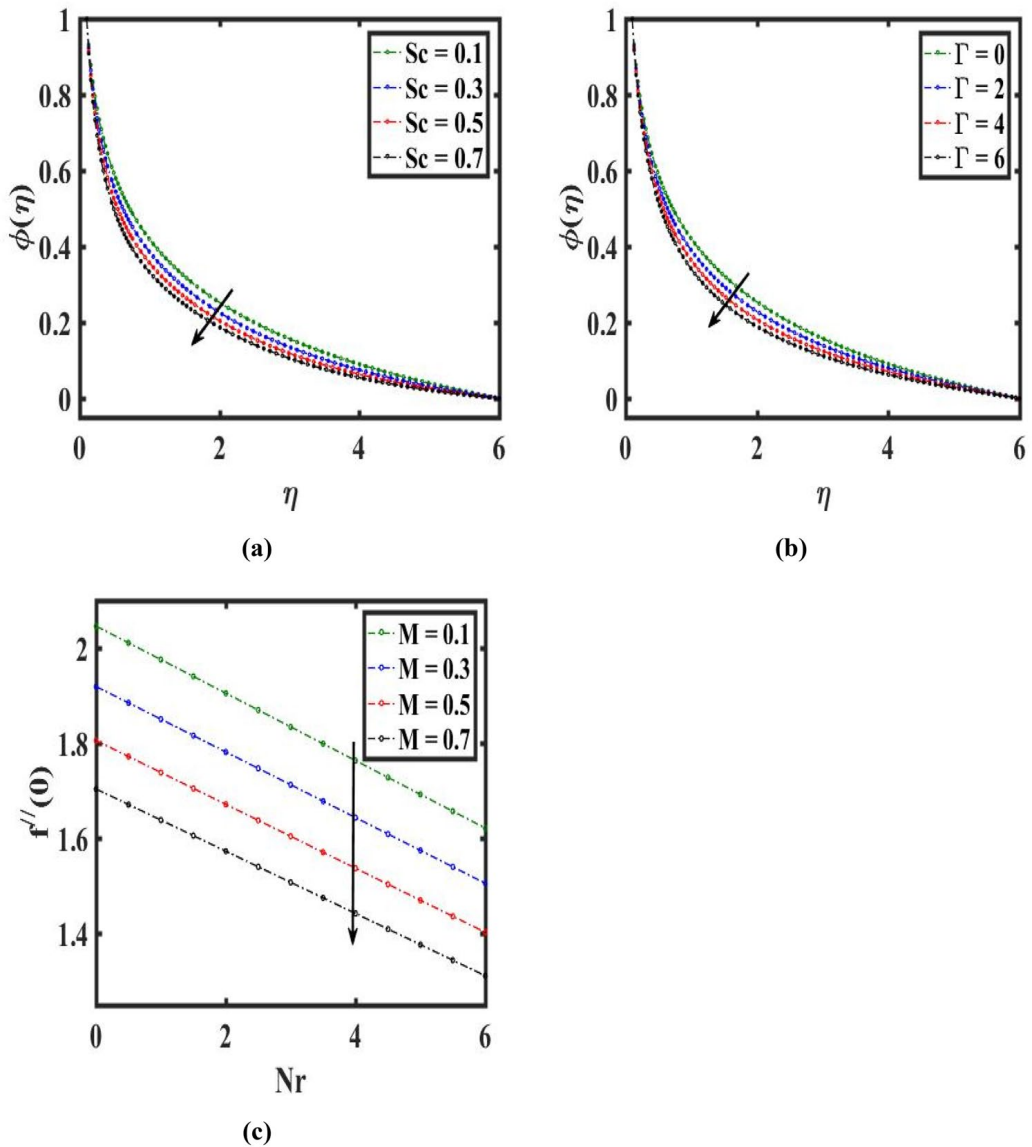
Effect of Schmidt number $Sc$, chemical reaction $\Gamma$, Hartmann number $M,$ and buoyance ratio $Nr$ on concentration $\phi (\eta )$ and velocity ${f}^{{{\prime}}{{\prime}}}(0)$.

**Figure 7:**
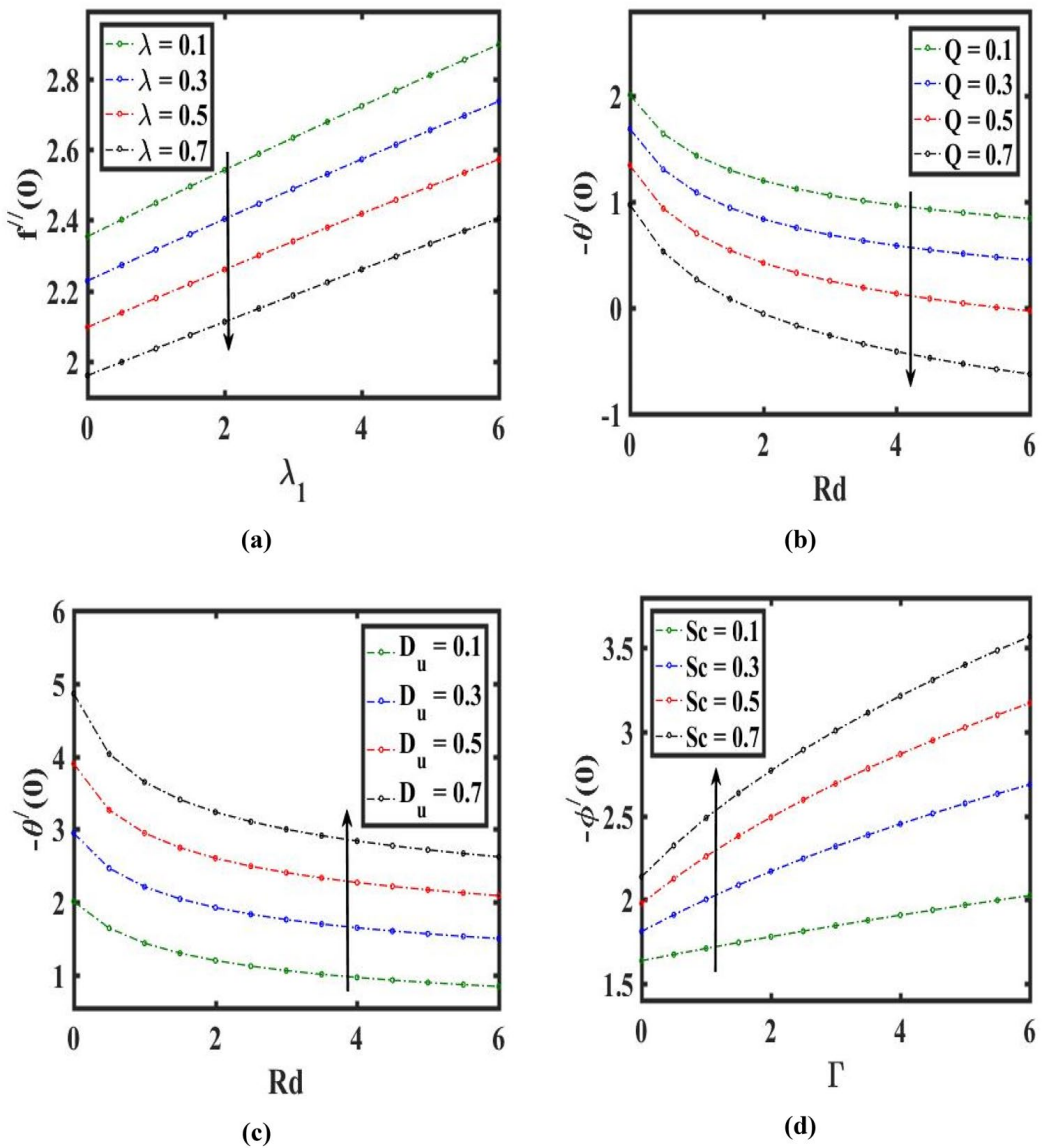
Influences of velocity ratio $\lambda$, mixed convection ${\lambda }_{1}$, heat generation $Q$, Dufour number ${D}_{u}$, radiation $Rd$, Schmidt number $Sc$ and chemical reaction $\Gamma$ on drag force ${f}^{{{\prime}}{{\prime}}}\left(0\right)$, heat transfer $-{\theta }^{{\prime}}(0)$ and mass transfer $-{\phi }^{{\prime}}(0)$.

The change of $\phi (\eta )$ through $(Sc)$ is represented in Fig. 6a. this graph noticed that $\phi (\eta )$ and its associated layer thicker are declines for climbs estimation of $(Sc)$. In addition, for raising the estimation of $(Sc)$. $\phi (\eta )$ feels decay due to it. The variability of the concentric distribution $\phi (\eta )$ with $(\Gamma )$ is revealed in Fig. 6b. As the amount of $(\Gamma )$ climbs the concentric of species in the boundary layered falls. Figure 6c highlights the influence of the drag coefficient along $(M)$ and $(Nr)$. Here, augmenting estimation of $(M)$ decay the $f^{\prime\prime}\left( 0 \right)$. Figure 7a displays the friction factor coefficient change towards $(\lambda )$ and $({\lambda }_{1})$. Here, a higher amount of $(\lambda )$ diminishes the $f{^{\prime}}{{\prime}}(0)$*.* The change in heat flux through $(Q)$ and $(Rd)$ is depicted in Fig. 7b. Here, $-\theta {^{\prime}} (0)$ reduces for enhancing estimation of $(Q)$ and falls for improving the estimation of $(Rd)$. The variation in heat flux towards $({D}_{u})$ and $(Rd)$ is illustrated in Fig. 7c. Here, $-\theta {^{\prime}} (0)$ acts as an escalating function of $({D}_{u})$ and diminishing function of $(Rd)$. The difference in mass flux through $(Sc)$ and $(\Gamma )$ is revealed in Fig. 7d. Here, $-\phi {^{\prime}} (0)$ upsurge for escalating estimation of $(Sc)$ and decays for higher in the amount of $(\Gamma )$.

## Concluding Remarks

A numerical study of the melting heat transport process in MHD flow of Cross fluid passing on a movable thinner needle is investigated. The influences of nonlinear heat radiative, Soret, and Dufour on mixed convection flow have been taken into consideration. Additional changes in the binary chemical reaction and thermal generation have also been studied. The core observations of the current study are summarized as follows:

- Large estimations of ${{\lambda}}_{1}$ and ${{N}}{{r}}$ yields the velocity field and thickness of the momentum boundary layer.
- Reverse behavior of the thermal field is analyzed in the frame of ${{{D}}}_{{{u}}}$ and ${{R}}{{d}}$.
- Qualitative impact of concentration and Sherwood number are reversed when ${{S}}{{c}}$ is enhanced.
- Coefficient of Skin fraction is decay via mixed convection parameters.
- Local heat flux is improved because of the Dufour effect.
- Local mass flux is an increasing function of the Schmidt number.

The RK-fourth order and shooting techniques could be applied to a variety of physical and technical challenges in the future^43–50^.

## Date availability

All data generated or analyzed during this study are included in this published article.

## List of symbols

$$u, v$$: Velocity components (m/s)
$$x,y$$: Cartesian coordinates (m)
$n$: Power law index
$$T$$: Fluid temperature (K)
$Q$: Heat generation parameter
${\rho }_{p}$: Density of nanoparticle
$${T}_{w}$$: Surface temperature (K)
$\mathrm{Re}$: Local Reynolds number
$\mu$: Generalized Newtonian viscosity (Pa s)
$$\sigma$$: Electric conductivity
$${U}_{w}$$: Stretching velocity (m/s)
$$k$$: Thermal conductivity (W/m K)
$${B}_{0}$$: Magnetic parameter
$$\psi$$: Stream function
${\lambda }_{1}$: Mixed convection parameter
$C$: Fluid concentration (kg/m^3^)
$a$: Needle thickness parameter
${C}_{w}$: Surface volume fraction (kg/m^3^)
$We$: Local Weissenberg number
$${T}_{\infty }$$: Ambient temperature (K)
$${D}_{u}$$: Dufour number
$${C}_{s}$$: Concentration susceptibility
$$\mathrm{Pr}$$: Prandtl number
$$\phi$$: Dimensionless concentration
$Nu$: Local Nusselt number
${\theta }_{w}$: Temperature ratio parameter
${K}_{r}^{2}$: Chemical reaction constant
$$Sr$$: Soret number
${C}_{f}$: Skin friction coefficient
$\lambda$: Velocity ratio parameter
${C}_{\infty }$: Ambient nanoparticle volume fraction (kg/m^3^)
$M$: Magnetic effect (kg/As^2^) Tesla
$${\nu }_{f}$$: Kinematic viscosity (m^2^/s)
$$Rd$$: Radiation parameter
$g$: Thermal expansion coefficient
$$\theta$$: Dimensionless temperature
$\Gamma$: Chemical reaction parameter
$\eta$: Dimensionless similarity variable
$$Sc$$: Schmidt number
$Nr$: Buoyancy ratio parameter
$${D}_{m}$$: Mass diffusivity
$$E$$: Activation energy
$${C}_{p}$$: Heat capacity
$\rho$: Density of fluid (kg/m^3^)
